# How Common is Essential Tremor? Update on the Worldwide Prevalence of Essential Tremor

**DOI:** 10.5334/tohm.632

**Published:** 2021-07-09

**Authors:** Elan D. Louis, Morgan McCreary

**Affiliations:** 1 Department of Neurology, University of Texas Southwestern, Dallas, TX, USA

**Keywords:** Essential tremor, epidemiology, prevalence, definition

## Abstract

**Background:**

Essential tremor (ET) is among the most prevalent movement disorders. Comprehensive reviews of disease prevalence were published in 1998 and 2010 but not since then. We reviewed the prevalence of ET in population-based epidemiological studies, derived a precise summary estimate of prevalence from these studies, and examined differences in prevalence across studies. We used two methods: a descriptive-analytical approach and a meta-analysis.

**Methods:**

A PUBMED search yielded 14 published papers since the 2010 review.

**Results:**

There were 42 population-based prevalence studies (23 countries and 6 continents). In a meta-analysis, pooled prevalence (all ages) = 1.33%, with statistically significant heterogeneity across studies (I^2^ = 99.3%, p < 0.0001). In additional descriptive analyses, median crude prevalence (all ages) = 0.4% and mean = 0.67%. Prevalence increased markedly with age, and especially with advanced age. In the meta-analysis, prevalence (age ≥ 65 years) = 5.79%, and in descriptive analyses, median crude prevalence (age ≥ 60–65) = 5.9% and mean = 8.0%. In the oldest age groups, median prevalence = 9.3%, with several studies reporting values >20%. The prevalence increased by 74% for every decade increase in age (p < 0.0001). Gender did not impact the prevalence of ET (p = 0.90).

**Discussion:**

Precise prevalence estimates are important because they form the numerical basis for public health initiatives and offer clues about underlying biological factors of mechanistic importance. The prevalence of ET among those age ≥ 65 is similar to that reported for Alzheimer’s disease in elders, suggesting that ET may be the most common neurodegenerative disease.

## Introduction

Essential tremor (ET) is among the most prevalent movement disorders, and it has been argued, one of the most common neurodegenerative diseases [1]. Patients with this disease, of whom there are an estimated 7 million in the United States alone [2], seek medical attention not only from neurologists but also from a range of primary care providers [3,4,5,6,7,8]. While many individuals with this disease seek medical attention, we know from population-based epidemiological studies, that there are many more cases in the population that have not been formally diagnosed or who have not sought medical attention for their tremor [9,10,11,12,13].

Establishing a precise prevalence estimate for ET is important. First, such an estimate is needed in order to gauge the need for public health initiatives aimed at preventing or resourcing the treatment of this disease. Second, estimates of disease prevalence within the population are critical in evaluating potential susceptibility genes in genetic research. Third, an understanding of the background level of occurrence of tremor in the population assists with the interpretation of phenotypic data in family studies. Fourth, it is important for those who are gauging the value of novel therapeutics to understand the size of the population with which they are dealing. Indeed, the study of any disorder begins with a reckoning and understanding of the countable number of cases.

One of the authors (E.D.L.) initially undertook a systematic review of the ET prevalence literature in 1998; there were 14 population-based prevalence studies [9]. In a follow-up review by the same author in 2010, the number of such studies had increased to 28 [14]. There has been no update in a decade. During that time, 14 new population-based studies have emerged. Furthermore, methodologies have continued to improve over time, with greater attention to details of case definition, for example. The purpose of this study is to review the prevalence of ET in population-based epidemiological studies, derive a precise summary estimate of prevalence from these studies, and examine differences in prevalence across studies. To address our aims, we used two methods: a descriptive-analytical approach and a meta-analysis.

## Methods

### Literature Review

In January 2021, we searched PUBMED for full scientific papers, going back to February 2009, which was the end of the time period covered in our 2010 paper [14]. In the first inquiry, we used two key word search terms, “prevalence” and “essential tremor”, and this yielded 164 published papers. The second inquiry used two key word search terms, “epidemiology” and “essential tremor”, and this yielded 392 published papers. The third inquiry used two key word search terms, “population” and “essential tremor”, and this yielded 224 published papers. Each of these papers was reviewed, and these combined searches yielded a total of 14 published papers that had not been reviewed in our 2010 paper [14].

### Statistical Analysis

We performed a meta-analysis using the meta and metafor packages in R [15,16,17].

Data were pooled based on the number of ET cases and the total population screened in each study. The pooled prevalence rate and 95% confidence intervals (CI) were estimated using a random effects model with inverse variance weighting, along with the event rates and estimated 95% CIs for each study. Cochran’s Q statistic was computed as a measure of between-study heterogeneity and I^2^, the percentage of variability due to between-study heterogeneity, was calculated. All plots were generated using the ggplot2 package in R [18].

## Results

### Introduction

One paper [19] that was included in the 2010 review was superseded by a follow-up study from the same group; [20] in the follow-up study, more extensive data were presented. Therefore, only the follow-up study is presented [20]. In addition, the 2010 review did not capture one paper [21]. Hence, this review includes 27 studies [10,11,12,13,22,23,24,25,26,27,28,29,30,31,32,33,34,35,36,37,38,39,40,41,42,43,44] that were covered in the prior review, one that was not captured in the prior review [21], and 14 new ones [20,45,46,47,48,49,50,51,52,53,54,55,56,57] for a total of 42 [10,11,12,13,20,22,23,24,25,26,27,28,29,30,31,32,33,34,35,36,37,38,39,40,41,42,43,44,45,46,47,48,49,50,51,52,53,54,55,56,57]. These 42 studies were from 23 countries across 6 continents (Asia = 18, Europe = 10, North America = 6, Africa = 5, South America = 2, Australian continent = 1) [10,11,12,13,20,21,22,23,24,25,26,27,28,29,30,31,32,33,34,35,36,37,38,39,40,41,42,43,44,45,46,47,48,49,50,51,52,53,54,55,56,57]. Several countries were represented by more than one study (USA = 5, China = 5, Spain = 4, Turkey = 4, Italy = 3, Israel = 3, India = 2, Nigeria = 2).

### Meta-Analysis

**Figure 1:**
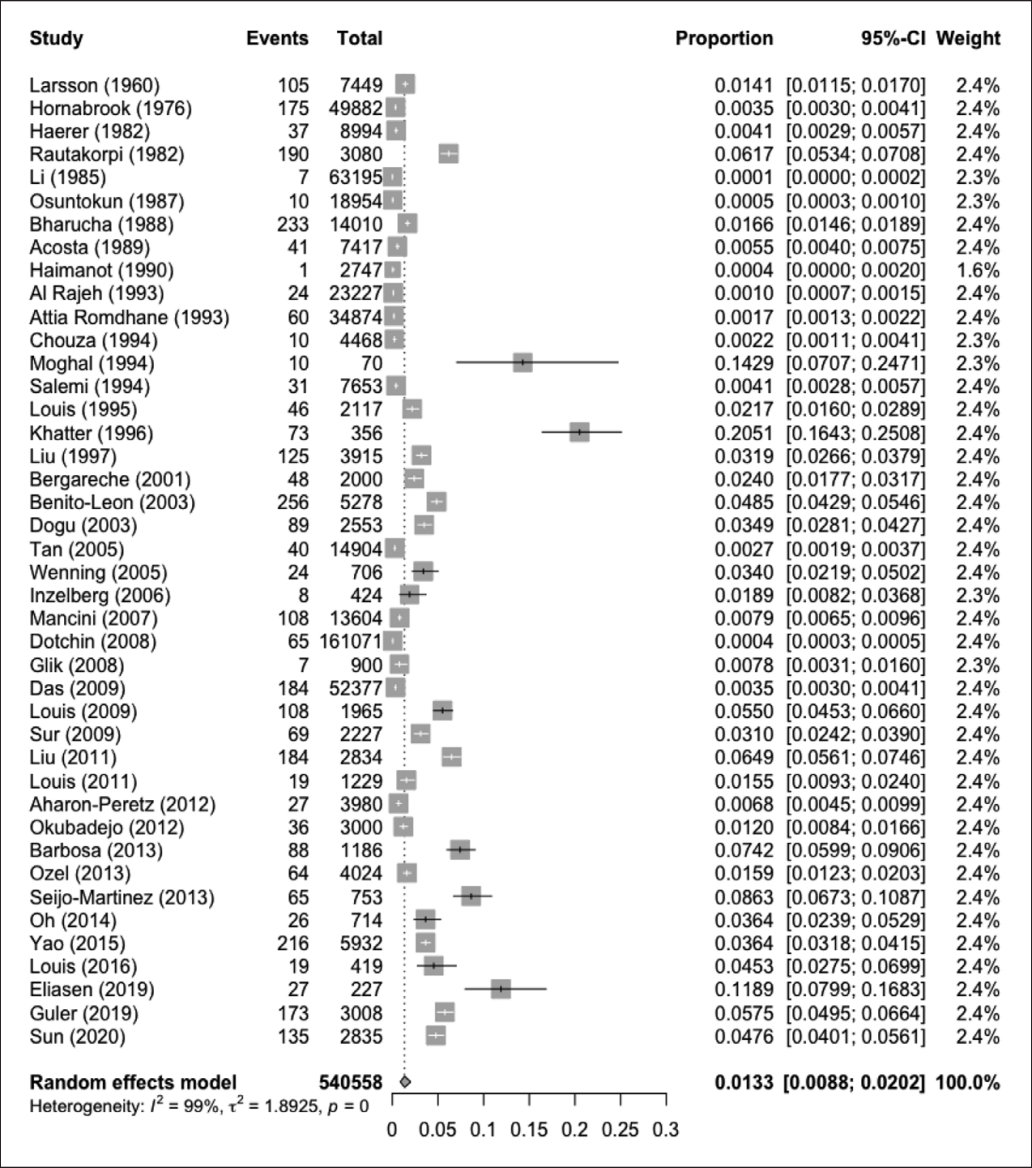
Forest plot of meta-analysis of the prevalence estimates from all 42 studies.

The 42 studies identified 3,263 ET cases from a total of 540,558 participants screened. When pooling all studies, the overall estimated prevalence of ET = 1.33% (95% CI = 0.88% - 2.02%), but there was considerable heterogeneity across studies (Q = 5729.34, I^2^ = 99.3%, p < 0.0001) (***Figure 1***).

We performed a number of additional analyses. First, subgroup analyses were performed to test for a potential difference between studies with an explicit definition of ET versus those without an explicit definition. A total of 36 studies used an explicit definition of ET while 6 studies did not have an explicit definition of ET. It was determined that the estimated pooled prevalence in studies with an explicit definition of ET was 2.00% (95% CI = (1.31%, 3.03%)) and the estimated pooled prevalence in studies without an explicit definition of ET was 0.10% (95% CI = (0.04%, 0.26%)). Furthermore, the data suggest that the pooled prevalence within these two groups were significantly different (Q = 30.39, p < 0.001). Despite separation of the two respective cohorts, considerable heterogeneity remained in the cohort with an explicit definition of ET (Q = 4860.21, I^2^ = 99.3%, p < 0.001) and the cohort without an explicit definition of ET (Q = 123.28, I^2^ = 95.9%, p < 0.001).

Second, we performed a meta-analysis of all studies with an explicit definition of ET while excluding the three studies with the greatest confidence interval lengths [42,43,55]. The resulting estimated prevalence of ET was found to be 1.65% (95% CI = (1.08, 2.52)). Considerable heterogeneity remained (Q = 4401.80, I^2^ = 99.3%, p < 0.001).

**Figure 2:**
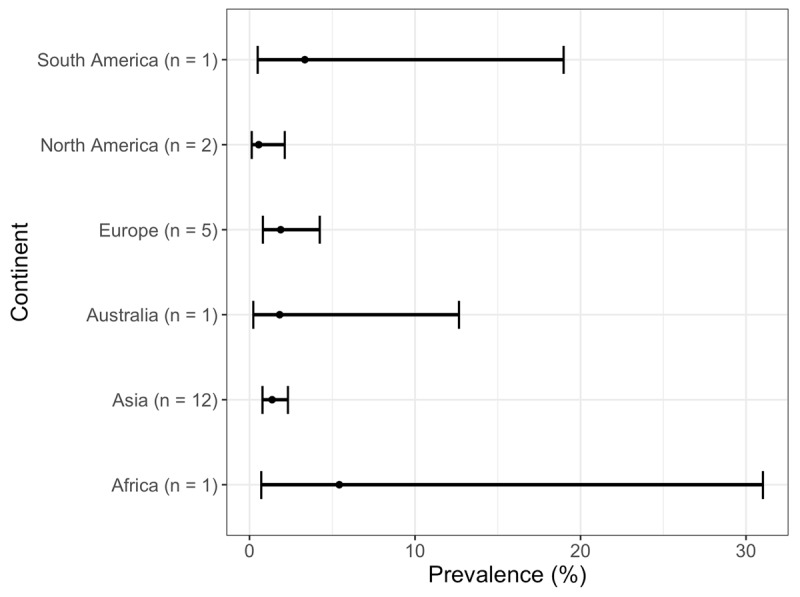
Prevalence of ET by continent.

Third, a meta-regression was performed to examine difference between continents, while controlling for age. Given that prevalence data stratified by age was not available for studies from all continents, the mean age of the cohort was used instead. Furthermore, the mean age of the cohort was centered and scaled to adjust the estimated prevalence reported for each continent to correspond to that of the average mean age (57.12 years old). The mean age of the cohort under investigation was available for a total of 22 of the 42 studies. ***Table 1*** presents the estimated prevalence and 99.2% CI by continent. The 99.2% CI was specified to adjust for the multiple comparison of 6 continents (i.e., 1 – 0.05/6 = 0.992). A plot of the estimated ET prevalence is shown (***Figure 2***). Based on the plot of the 99.2% CIs, we do not see a difference in the prevalence of ET between the 6 continents. However, it must be noted that only a single study reported the mean age of the cohort under investigation for each of the continents of Africa, Australia, and South America. This resulted in much wider confidence intervals for the prevalence of ET from these continents relative to Asia, Europe and North America. The heterogeneity that remained between trials was significant (Q = 717.20, I^2^ = 97.91%, p < 0.0001).

**Table 1:** Estimated prevalence of ET by Continent for a cohort with an average age of 57.12 years old.


CONTINENT	NUMBER OF STUDIES	ESTIMATED PREVALENCE	99.2% CI

Africa	1	5.42%	(0.73, 31.02)

Asia	12	1.36%	(0.79, 2.33)

Australia	1	1.82%	(0.24, 12.67)

Europe	5	1.88%	(0.82, 4.26)

North America	2	0.56%	(0.14, 2.13)

South America	1	3.33%	(0.51, 18.99)


CI = confidence interval.

**Figure 3:**
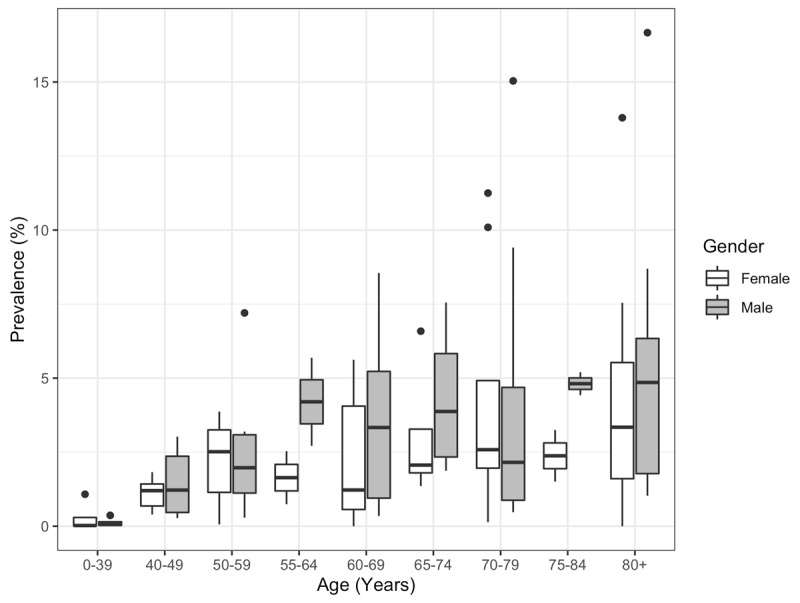
Prevalence by age group and gender. Data from 15 studies.

**Figure 4:**
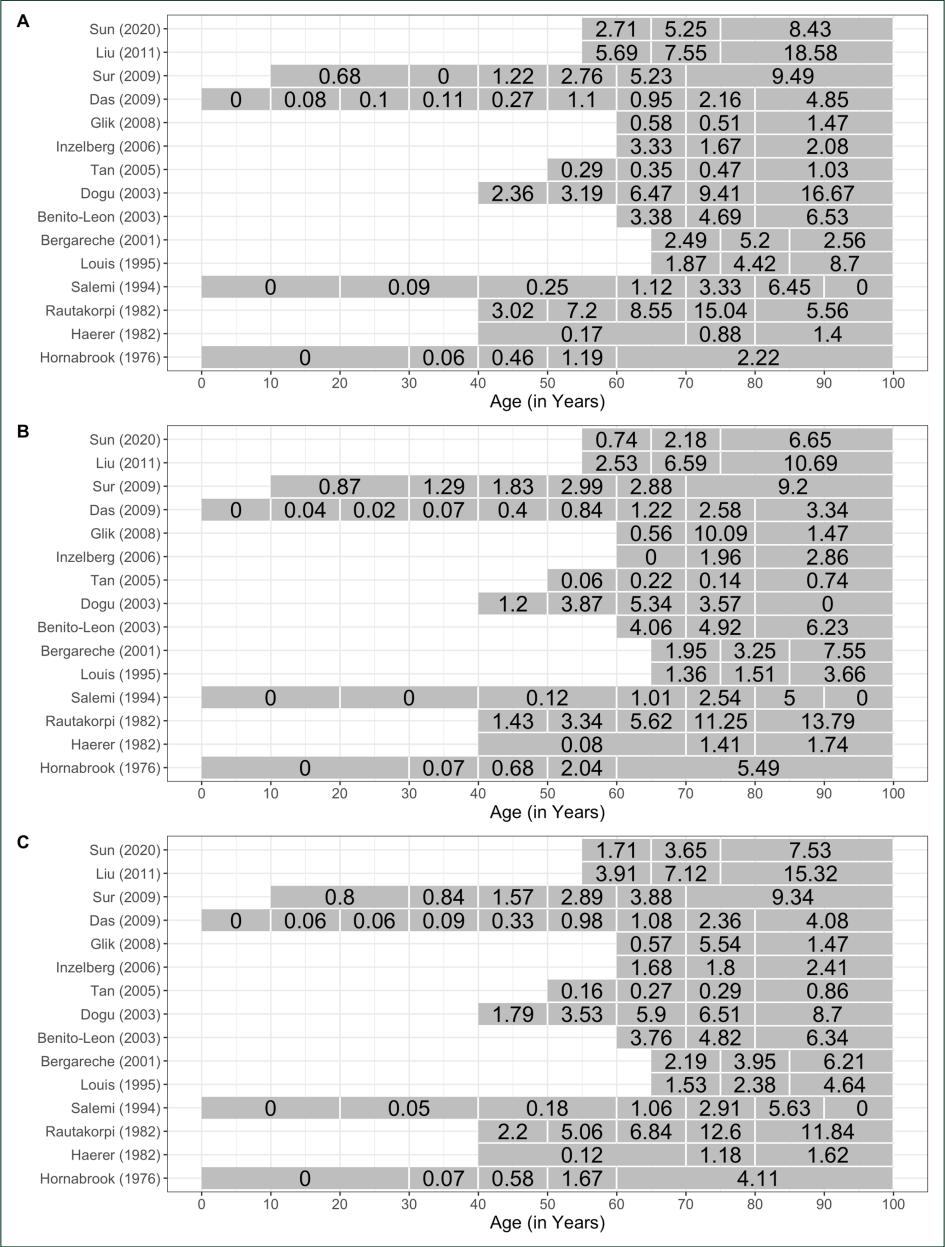
Prevalence by study and age group in males **(a)**, females **(b)**, and both genders **(c)**.

Fourth, prevalence data stratified by age and gender were available for 15 of the 42 studies. The variability of the prevalence data by age group and gender is shown in ***Figure 3*** and the raw prevalence by age strata for each study are shown in ***Figure 4***. A meta-regression model was implemented to test for a change in prevalence as age increases, controlling for gender. In order to construct such a model, those subjects aged 0–39 years served as the reference category and categories 40–49 up to 80 years of age were ordered numerically relative to the 0–39 category. That is, 40–49 was assigned a 1, 50–59 a 2, 60–69 a 3, and so on up to 80 being assigned a 5. Furthermore, for those studies reporting prevalence beginning at half-decades (e.g., 55–64), the numerical value was recorded as the average of the two decades. Lastly, those studies reporting data that completely spanned two decades, other than those in the 0–39 strata, were excluded (e.g., 40–59). Based on the results of this analysis, it is estimated that the prevalence increases by a factor of 1.74, or 74%, for every decade increase in age (p < 0.0001). Additionally, gender was not found to impact the prevalence of ET (p = 0.90). The heterogeneity among studies remained high with a Q value of 989.69 (p < 0.0001) and an I^2^ of 89.79%.

Fifth, a subgroup analysis was performed to estimate the prevalence in those subjects ≥65 years of age. A total of 14 studies used an explicit definition of ET and reported data for subjects ≥65 years of age. Based on this cohort, the pooled prevalence for those ≥65 years of age was 5.79% (95% CI = (4.14%, 8.05%), Q = 353.02, I^2^ = 96.3%, p < 0.001). However, based on the Q statistic, considerable heterogeneity remained between studies.

In each analysis, considerable heterogeneity is present across studies. Therefore, the conclusions one may draw from pooling the studies should be interpreted with caution and this justifies the descriptive analytic approach and narrative review conducted in the remainder of this manuscript.

### Descriptive Analytic Approach

#### Pertinent Methodological Issues That Arise When Interpreting Data from Prevalence Studies

There is considerable heterogeneity of prevalence estimates in ET, a difference that exceeds that seen for many other neurological disorders; differences in methods of ascertainment, methods of case evaluation, case definition, and demographics of study population account for a sizable amount of this heterogeneity. We review further now.

As reviewed previously [14], there are several factors that could explain the large difference that is observed in prevalence estimates of ET. Data presented in population-based studies indicate that only a small percentage of ET cases may seek medical attention for their tremor, with some such estimates as low as 0.0% [25], 0.5% [12], and 2.8% [47] of identified cases seeking medical attention, particularly if these cases live in rural or medically underdeveloped areas [12,25,47]. As a result, studies that ascertain their ET cases from treatment settings underestimate the true prevalence of ET. The 42 studies that we review here are all population-based studies; none ascertained their cases from clinics. Hence, this issue is moot with respect to the current set of studies.

The age composition of the sampled population is an important factor that can influence the estimate of disease prevalence, and the majority of studies show that the prevalence of ET rises with age [10,11,13,20,21,29,31,32,33,36,37,39,40,41,44,45,46,47,48,49,50,51,53,54,55,56,57]. Developing countries and countries that have higher birth rates will have an age structure that is younger than is that of their counterparts that do not have these characteristics. For example, in a study in India, 32.4% of the sampled population was younger than 20 years of age and 51.1% was younger than 30 years of age [20]. In a study in Bangladesh, 80.5% of the sampled population was younger than 40 years of age [45]. In a study in Nigeria, 51.2% of the sampled population was younger than age 25 years and 71.0% was younger than age 30 years [47]. In a study in New Guinea, 66.9% of the sample population was under the age of 30 years [31]. The crude prevalence (all ages) in these studies tends to be low: 0.35% [20] and 0.4% [31].

A third methodological issue is whether individuals in prevalence studies were each examined or whether they were first screened using a questionnaire or another screening instrument (e.g., a screening spiral) and then examined based on a positive response to that screening process (***Table 2***). It has been demonstrated that screening questionnaires for ET have modest rather than high sensitivity (generally in the 60–70% range) [58,59], and that sensitivity is lowest among milder cases, that is, the types of ET cases typically ascertained in population-based rather than clinic-based studies. The larger majority of studies has relied on screening instruments rather than universal examination, although there are examples of the latter [10,51]. There are a number of studies that relied on screening instruments but which also provided data on the sensitivity of their screening instrument [11,13,39,41,47], thereby allowing investigators to calculate an estimated prevalence that approximates a study design in which all participants had received a neurological examination. Studies that used either this approach or which used universal examinations have tended to provide higher estimates of prevalence [10,11,13,31,39,40,41,42,43]. One additional issue is that neurologists and, even more so, movement disorder neurologists, are more likely to recognize and distinguish ET from other forms of tremor when they examine patients and studies that employ them are more likely to provide valid estimates of prevalence.

**Table 2:** Crude prevalence of ET in 42 population-based studies.


AUTHOR	YEAR	COUNTRY	PREVALENCE (%)	AGES	EXAMINED ALL SUBJECTS (WHOM)

Li [24]	1985	China	0.01	All	No

Dotchin [25]	2008	Tanzania	0.04	All	No

Haimanot [26]	1990	Ethiopia	0.04	All	No

Osuntokun [23]	1987	Nigeria	0.05	All	Unclear from study description

Al Rajeh [27]	1993	Saudi Arabia	0.2	All	No

Attia Romdhane [28]	1993	Tunisia	0.2	All	No

Chouza [30]	1994	Uruguay	0.2	All	No

Tan [29]	2005	Singapore	0.3	≥50	No

Das [20]	2009	India	0.35	All	No

**Hornabrook** [31]	**1976**	**New Guinea**	**0.4**	**All**	**Yes (field officer)**

**Salemi** [32]	**1994**	**Italy**	**0.4**	**All**	**Yes (neurologists)**

Haerer [33]	1982	USA	0.4	≥40	No

**Inzelberg** [21]	2006	**Israel**	**0.5**	**≥65**	**Yes (neurologists)**

Acosta [34]	1989	Spain	0.6	All	Yes (nurses, General practitioners)

Aharon-Peretz [48]	2012	Israel	0.7	≥51	No

**Glik** [35]	**2009**	**Israel**	**0.8**	≥**65**	**Yes (neurologist)**

**Mancini** [36]	**2007**	**Italy**	**0.8**	≥**41**	**Yes (General practitioners)**

**Okubadejo** [47]	**2012**	**Nigeria**	**1.2 [1.2]**	**All**	**No but information provided on sensitivity of screening instrument**

Larsson [12]	1960	Sweden	1.4	All	No

Louis [45]	2011	Bangladesh	1.6	>18	Yes (using spirals)

Ozel [49]	2006	Turkey	1.6	18-60	No

Bharucha [37]	1988	India	1.7	All	No

Eliazen [55]	2019	Faroe Islands	2.9	≥40	No

**Sur** [38]	**2008**	**Turkey**	**3.1**	≥**18**	**Yes**

Wenning [44]	2005	Austria	3.4	50–89	Yes (neurologists, geriatricians, other medical specialists)

**Dogu** [10]	**2003**	**Turkey**	**3.5**	≥**40**	**Yes (neurologists)**

**Oh** [52]	**2014**	**Korea**	**3.6**	**≥65**	**Yes**

Yao [53]	2015	China	3.6	≥45	No

**Louis** [13]	**1995**	**USA**	**2.2 [3.9]**	≥**65**	**No but information provided on sensitivity of screening instrument**

**Louis** [54]	**2016**	**USA**	**4.5**	≥18	**Yes**

**Sun** [57]	**2020**	**China**	**4.8**	≥55	**No**

**Louis** [41]	**2009**	**USA**	**5.5**	≥**65**	**Yes (handwriting samples reviewed by movement disorder specialist)**

Guler [56]	2019	Turkey	5.75	≥18	No

**Bergareche** [39]	**2001**	**Spain**	**2.4 [6.4]**	≥**65**	**No but information provided on sensitivity of screening instrument**

Liu [46]	2011	China	6.5	≥55	No

Liu [22]	1997	China	6.5	≥50	Yes (neurologists)

**Benito-Leon** [40]	**2003**	**Spain**	**4.9 [7.0]**	≥**65**	**No but information provided on sensitivity of screening instrument**

Barbosa [50]	2013	Brazil	7.4	≥64	No

**Seijo-Martinez** [51]	**2013**	**Spain**	**8.6**	**≥65**	**Yes**

**Rautakorpi** [11]	**1982**	**Finland**	**6.2 [9.7]**	≥**40**	**No but information provided on sensitivity of screening instrument**

**Moghal** [42]	**1994**	**Canada**	**14.3**	≥**65**	**Yes**

**Khatter** [43]	**1996**	**USA**	**20.5**	≥**65**	**Yes (not specified)**


Studies are ordered from lowest to highest prevalence (%). All values in brackets account for the sensitivity of the initial screening process (i.e., values are higher because they include an estimate of the number of false negatives). In bold are the studies that: (1) either examined all subjects or provided information on screening questionnaire and (2) provided separate age-stratified estimates of prevalence among elderly aged 60 and older.

Finally, the deﬁnition of ET is critical. While most prevalence studies reported in our 2010 paper defined ET, we reported that six did not [14]. In the 14 studies that have been published since that report, all have provided explicit definitions of ET [20,45,46,47,48,49,50,51,52,53,54,55,56,57], which represents an improvement in methodology over time. However, we had noted in the earlier report that the large majority of studies used deﬁnitions that either did not specify the examination that was performed on participants or the minimal severity of tremor that was required to qualify for a diagnosis [14]. In the 14 studies that have been published since the last report, a number do not report the specific examination maneuvers used to assess tremor [20,47,49,52]. Furthermore, many studies have used Consensus criteria for ET [60], which were not designed for population-based studies, and do not allow investigators to systematically distinguish enhanced physiological tremor from ET.

#### Arriving at a More Refined Estimate of Prevalence

The issues that were discussed above can be used in order to derive a more refined estimate of disease prevalence. All of the studies we have included are population-based, and these provide more valid estimates than clinic-based series. One may see that prevalence ranges from 0.01% to 20.5% (***Table 2***), although studies were conducted on samples with very different age cut-offs and age structures. As discussed above, it is preferable to select studies in which each subject was examined or in which data on the sensitivity of the screening questionnaire in their population may be used to make adjustments for false negative screens. Using this approach, there are three studies that provide data across the life span (***Table 2***). These were conducted in New Guinea [31], Italy [32] and Nigeria [47]. Using this strategy, the calculated prevalence of ET (all ages) was 0.4% [31], 0.4% [32] and 1.2% [47]. The mean is 0.67% and the median is 0.4%.

The crude prevalence in older age groups, age 60–65 and older, ranges from a low of 0.5% to 26.1% (refer to unbracketed and bracketed values in ***Table 3***) with the median = 5.9% and mean = 8.0%. If one removes those studies that are potential outliers, that is, the two studies with the lowest and the two with the highest prevalence estimates, median = 5.9% and mean = 6.9%, ***Table 3***). Furthermore, the prevalence continues to rise with age, with crude prevalence estimates in the oldest age groups (80s, 90s and older) ranging from 1.2% to 42.9% (***Table 3***), with the mean in the highest age group = 11.4% and median = 9.3%.

**Table 3:** Crude prevalence of ET (older age categories) in population-based prevalence studies.


AUTHOR	YEAR	COUNTRY	PREVALENCE ≥60 YEARS* (%)	PREVALENCE IN OLDEST AGE GROUP (%)

Inzelberg [21]	2006	Israel	0.5 (≥65 years)	1.2 (≥80 years)

Glik [35]	2009	Israel	0.8 (≥65 years)	1.5 (≥80 years)

Mancini [36]	2007	Italy	2.1 (≥61 years)	3.3 (81–90 years) and 3.6 (≥90 years)

Salemi [32]	1994	Italy	2.3 (≥60 years)	5.4 (≥80 years)

Oh [52]	2014	Korea	3.6 (≥65 years)	1.4 (≥80 years)

Louis [13]	1995	USA	2.2 [3.9] (≥65 years)	4.6 [8.4] (≥85 years)

Hornabrook [31]	1976	New Guinea	4.1 (≥60 years)	No data

Louis [54]	2016	USA	5.1 (≥ 65 years)	10.9 (≥80 years)

Louis [41]	2009	USA	5.5 (≥65 years)	9.9 (85–94 years), 21.7 (≥95 years)

Dogu [10]	2003	Turkey	6.3 (≥60 years)	8.7 (≥80 years)

Bergareche [39]	2001	Spain	2.4 [6.4] (≥65 years)	9.7 [12.9] (≥85 years)

Benito-Leon [40]	2003	Spain	4.8 [7.0] (≥65 years)	7.3 [10.6] (≥85 years)

Seijo-Martinez [51]	2013	Spain	8.6 (≥65 years)	11.2 (≥85 years)

Sur [38]	2008	Turkey	11.5 (≥61 years)	9.3 (≥71 years)

Moghal [42]	1994	Canada	14.3 (≥65 years)	No data

Rautakorpi [11]	1982	Finland	9.0 [15.6] (≥60 years)	11.8 [20.7] (≥80 years)

Khatter [43]	1996	USA	20.5 (≥65 years)	No data

Okubadejo [47]	2012	Nigeria	26.1 (≥65 years)	42.9 (≥85 years)


Table includes studies: (1) either examined all subjects or provided information on screening questionnaire and (2) provided separate age-stratified estimates of prevalence among elderly aged 60 and older. Studies are ordered from lowest to highest prevalence (%) in the ≥60 year age stratum. All values in brackets account for the sensitivity of the initial screening process (i.e., values are higher because they include an estimate of the number of false negatives). * In some studies, age stratum was ≥60 while in others (as indicated), it was ≥61 or ≥65.

#### Additional Patterns in Prevalence

##### Age

The majority of studies provide age-stratified data [10,11,13,20,21,29,31,32,33,36,37,38,39,41,44,45,46,47,48,49,50,51,53,54,55,56,57]. The prevalence of ET rises considerably with age, and especially during advanced age [10,11,13,20,21,31,32,35,38,39,40,41,45,46,47,48,49,50,51,53,54,55,56,57], thereby indicating that age and advanced aging is a risk factor for ET. In many studies, this increase is observed to be exponential during advanced age, a feature that is present in a variety of other neurodegenerative diseases such as Alzheimer’s disease and Parkinson’s disease [61]. There are limited data on the prevalence of ET among oldest old. In numerous studies, the size of the age strata diminishes markedly during the ninth or tenth decades of life, making these estimates less stable, and in some studies there is a paradoxical decrease in prevalence in these strata [52]. By contrast, a larger number of studies show a marked increase, with a number of studies reporting values in excess of 20% during the ninth and tenth decade of life [11,41,47].

ET is not a disease exclusively of adults; indeed, the disease may begin in childhood [62,63,64]. The majority of these young-onset cases are familial [65,66]. There are few population-based prevalence studies that have sampled children. In these, the crude prevalence in children has been reported as 0.0% [31,32,47] and 0.07% (up to age 19) [20], indicating that on a population-level, the prevalence of ET in this age group is low.

##### Ethnicity

Ethnic differences in the population prevalence of ET, if identified, could reﬂect differences in the prevalence of ET susceptibility genes or could reﬂect differences in exposure to environmental risk factors for ET [67]. There are limited data on such ethnic differences in ET. There are a few studies that have directly compared racial or ethnic groups. For example, a study in New Guinea reported differences in the prevalence of ET in populations that were deﬁned by different languages – a high prevalence of ET in villagers living in the Bena Bena and Kamano populations, and no ET cases among the Gimi or Yagaria [31]. A study in Singapore compared the prevalence of ET in Singaporean Chinese, Malays, and Indians, and reported a marginally higher prevalence in Indians compared to Chinese; no Malays were identiﬁed with ET. A study in Copiah county, Mississippi, USA reported a nonsigniﬁcant trend in which the prevalence of ET was higher in whites than African–Americans [33]. That study used on a screening questionnaire, however, which may have biased results toward lower prevalence among individuals with lower educational attainment [33]. Similarly, a study in northern Manhattan, USA, reported a nonsigniﬁcant trend in which the prevalence was higher in whites than African–Americans; that study similarly relied on an initial screening questionnaire [13]. By contrast, a study that re-sampled the same population several years later, and which did not rely on a screening questionnaire, reported a signiﬁcantly lower prevalence of ET among whites [41]. Clearly, more data are needed.

To further assess potential ethnic differences, one may also compare prevalence studies that sampled different ethnic groups in different regions of the same country. Thus, a study in the Basque region of Spain [39] provided estimates of prevalence that were similar to those provided in a study in Madrid, Spain [40]. One may contrast this with a study in the Parsi community of Bombay, India [37], which noted a higher prevalence than a study largely of Hindus in West Bengal, India [20]. These types of comparisons, however, are fraught with potential problems because lack of uniformity in study design, for example, the use of different screening protocols and the use of different definitions of ET, could explain differences.

To try to remedy this issue, one may compare studies that sampled different populations and ethnic groups but which used similar or identical study protocols. For example, population-based prevalence studies in Turkey, Arabs in Israel, and Basques in Spain did not rely on screening questionnaires and all used the same examination and a similar deﬁnition of ET. The crude prevalence of ET in these studies was 0.5% - 0.8% (≥65 years of age in Arabs in Israel) [21,35], 6.3% (≥60 years of age in Turkey) [10], 6.5% (≥61 years of age in Turkey) [38], 6.4% (≥65 years of age in Basques in Spain) [39], 8.6% (Arosa Island, Spain) [51]; these data suggest that there may be regional or ethnic differences in the prevalence of ET.

##### Gender

In the 2010 paper [14], we noted that of the 28 population-based prevalence studies of ET, nine (32.1%) did not provide gender-stratified data and a tenth study only reported a single ET case (i.e., the prevalence was extremely low). Of the remaining 18 studies, six (33.3%) noted a statistically higher prevalence among men (male: female ratios = 1.43, 1.50, 1.64, 1.65, 1.90, and 2.26:1) [11,13,25,29,36,43], and one (5.9%) reported a statistically higher prevalence among women (male: female ratio = 0.39:1) [31]. In the 14 additional studies published since then, 13 provided gender-specific data and two of these thirteen reported a significantly higher prevalence in men (male: female ratios = 1.67:1 and 1.84:1) [46,57]. In summary, of the 17 pre-2010 studies we include in this analysis and the 13 additional studies with gender-specific data that we now review (total number of studies = 30), eight (26.6%) reported a higher prevalence in men, one (3.3%) reported a higher prevalence in women, and 21 (70.0%) reported no difference between genders. Hence, the majority of studies (70%) demonstrate no gender difference.

##### Isolated Head tremor

Several studies in our 2010 paper reported data on the proportion of ET cases with isolated head tremor [13,25,31,32,40]. These data indicate that isolated head tremor was generally uncommon (0.0%, 1.6%, 3.2%) [13,32,40], although in two studies reached values of 9.1% [31] and 20.0% [25]. One methodological comment about the latter two studies is that the full extent of the assessment of upper limb tremor was not specified (i.e., it is unclear to what extent kinetic tremor was fully explored and with what range of different maneuvers), so that it is not certain whether some of these cases could actually have exhibited mild kinetic tremor, if they had been examined in full detail. Second, in those two studies, the neurological examination was not performed by a movement disorder neurologist, but rather, by a field officer, raising the possibility that some of the cases with isolated head tremor actually had cervical dystonia rather than ET; this is a frequent source of diagnostic misclassification [68,69,70]. In the 14 more recent studies, methodologies have advanced, and isolated head tremor was specifically noted to be present or absent in 7 of these studies. In these seven studies, the prevalence of isolated head tremor was as follows: 0.0% [47,48,55,57], 0.6% [56], 1.6% [49], 4.6% [51]. Overall, from the studies reviewed in our 2010 paper and from the more recent studies, the bulk of evidence indicates that the prevalence of isolated head tremor likely ranges between 0% and 5%. This being said, there are data which suggest that head tremor, if searched for carefully enough, may be more common than suspected. In a study of 241 first-degree relatives of ET cases, none of whom met diagnostic criteria for ET based on presence or severity of upper limb tremor, 26 (10.8%) exhibited an isolated, transient head tremor [71]. This fleeting tremor was noted during a deep phenotyping effort in which all individuals underwent a detailed videotaped tremor evaluation that was reviewed by a movement disorder neurologist. It is possible that the full extent of transient, mild, isolated head tremor in ET may be under-appreciated [72].

##### Previously Undiagnosed ET

ET cases who are living in communities may have mild tremor that does not prompt them to seek medical attention. Numerous studies provided data on the percentage of identified cases who were undiagnosed prior to the prevalence survey. These percentages are as follows: 100% (Tanzania) [25], 97.2% (Nigeria) [47], 97.1% (Finland) [11], 96.3% (Faroe Islands) [55], 92.8% (Turkey) [38], 92.2% (Turkey) [49], 91.0% (Turkey, Brazil) [10,50], 90.0% (Singapore) [29], 87.7% (Spain) [51], 79.7% (Spain) [40], 78.9% (USA) [54], and 59.5% (Turkey) [56]. These countries, which represent a broad socioeconomic range, indicate that the large bulk of population-dwelling ET cases are not seeking medical attention for their tremor and remain undiagnosed at the time of prevalence surveys. These data underscore for researchers that many ET patients do not engage with the health system.

## Discussion

Since the 2010 review, 14 additional population-based prevalence studies have been published [20,45,46,47,48,49,50,51,52,53,54,55,56,57], adding to those that had been published previously. In total, the 42 population-based prevalence studies of ET that we review here ascertained cases from a broad range of settings across 6 continents and 23 countries [10,11,12,13,20,21,22,23,24,25,26,27,28,29,30,31,32,33,34,35,36,37,38,39,40,41,42,43,44,45,46,47,48,49,50,51,52,53,54,55,56,57]. Although limited in its external validity by the significant heterogeneity across studies, a pooled estimate yielded an overall prevalence (all ages) = 1.33% (95% CI 0.88%, 2.02%). In an additional descriptive analysis, we observed that among studies that either directly examined each individual or published data on the sensitivity of their screening questionnaire, the median prevalence of ET, including all ages, was 0.4%, and the mean was 0.67%. We also observed a marked increase in prevalence with age. In the meta-analysis, the prevalence among those age ≥65 years = 5.79%, and in descriptive analyses, the median crude prevalence among individuals age ≥60–65 = 5.9%. We estimated that the prevalence increases by a factor of 1.74, or 74%, for every decade increase in age (p < 0.0001).

The prevalence studies we reviewed used combinations of screening questionnaires and/or in-person neurological examinations to ascertain ET cases. By contrast, the use of videotaped neurological examinations allows for repeated evaluation of subtle tremors, especially if viewed by a movement disorders neurologist. A recent study, which incorporated such a granular phenotyping of study subjects, observed transient isolated head tremor in a substantial proportion of relatives of ET cases, but of even greater interest, is that such tremor was detected in 2.6% of controls, suggesting that the prevalence of ET reported in traditional studies may be too low and that the real prevalence could be 60 – 80% higher [71].

The prevalence estimates we observe in ET serve to again confirm that this disease is very common; in fact, ET is often viewed as the most prevalent movement disorder among adults [9,14]. A population-based study in Italy of individuals ranging from 50–89 years of age [44] directly compared the prevalence of ET with that of other movement disorders. They reported that the prevalence of ET (3.1%) was higher than those of primary dystonia (0.8%), secondary dystonia (1.1%), tics (0.4%), and chorea (<0.2%) [44]. However, the prevalence of restless legs syndrome, 10.8%, was reportedly greater than that of ET [44]. A variety of conditions, including positional discomfort, cramps and local leg pathology can mimic restless legs syndrome, thereby greatly inflating prevalence estimates [73], and that study did not utilize the International Restless Legs Study Group diagnostic criteria, published in 2003 [74], also likely inflating the estimate of that condition. Nonetheless, studies that have used these criteria generally report estimates of the prevalence of restless legs syndrome that are higher than that reported for ET [75]. In most studies, the prevalence of ET is markedly higher than that of Parkinson’s disease [19,22,27,28,34,41,42,43]. The prevalence of ET among those age 65 and older is similar to that of Alzheimer’s disease in elders (median = 4.8%) [76]. Given the evidence that ET is likely degenerative [77,78], this would mean that ET was the first or second most common neurodegenerative disease.

Age is a clear risk factor for ET, as numerous studies report a marked age-associated rise in prevalence [10,11,13,20,21,31,32,35,38,39,40,41,45,46,47,48,49,50,51,53,54,55,56,57]. The increase with age is not linear, and seems to accelerate in advanced age. Values in excess of 20% are observed during the ninth and tenth decade of life [11,41,47]. Several studies suggest the presence of ethnic differences, however, the data are not conclusive and additional data are needed. No differences in ET prevalence between continents was discovered. However, published prevalence data from Africa, Australia, and South America with summary statistics of the distribution of age within the cohorts under investigation were sparse and contributed to the inability to precisely estimate the prevalence of ET in these continents. The majority of studies, 70%, demonstrate no gender difference. The meta-analysis did not reveal a gender difference either.

Data on overall prevalence of disease and the prevalence among different patient subgroups is important. Such data form the numerical basis for planned public health initiatives. They also provide clues about the existence of underlying demographic and biological factors of possible mechanistic importance.
